# Covid-19: Lessons for Mental and Brain Health

**DOI:** 10.1192/j.eurpsy.2022.47

**Published:** 2022-09-01

**Authors:** P. Falkai

**Affiliations:** Ludwig-Maximilians-Univeritiät Munich, Department Of Psychiatry And Psychotherapy, Munich, Germany

## Abstract

Psychiatry is facing major challenges during times of a pandemic as illustrated by the current COVID-19 pandemic. The challenges involve its actual and perceived role within the medical system, in particular how psychiatric hospitals can maintain their core mission of attending to the mentally ill while at the same time providing relief to general medicine. Since psychiatric disorders are the top leading causes of global burden of disease, we need to strengthen mental health care in the wake of the massive onslaught of the pandemic. While nobody can deny the need to act decisively and swiftly and ramp up intensive care readiness, we believe that the immediate availability of psychiatric care is just as important. In order to provide the best possible treatment conditions for people suffering from mental illness but as well for those suffering from the immediate pandemic’s consequences such as isolation, reduced social interaction etc. instant and comprehensive provision is inevitable.

**Disclosure:**

No significant relationships.

